# The Inheritance of Hearing Loss and Deafness: A Historical Perspective

**DOI:** 10.3390/audiolres14010010

**Published:** 2024-01-26

**Authors:** Alessandro Martini, Andrea Cozza, Valerio Maria Di Pasquale Fiasca

**Affiliations:** 1 Padova University Research Center “International Auditory Processing Project in Venice (I-APPROVE)”, Department of Neurosciences, University of Padua, 35128 Padua, Italy; 2 Department of Cardiac, Thoracic, Vascular Sciences and Public Health, University of Padua, 35128 Padua, Italy; 3 Section of Otorhinolaryngology—Head and Neck Surgery, Department of Neurosciences, University of Padua, 35128 Padua, Italy

**Keywords:** inheritance, genetics, epigenetics, hearing loss, deafness, deaf-mutism, consanguinity

## Abstract

If the term “genetics” is a relatively recent proposition, introduced in 1905 by English biologist William Bateson, who rediscovered and spread in the scientific community Mendel’s principles of inheritance, since the dawn of human civilization the influence of heredity has been recognized, especially in agricultural crops and animal breeding. And, later, in familial dynasties. In this concise review, we outline the evolution of the idea of hereditary hearing loss, up to the current knowledge of molecular genetics and epigenetics.

## 1. Introduction

The interest in genetics is particularly lively due to the recent finding of a “genetic” contributory cause to COVID-19 deaths [1,2].

The term “genetics” is a relatively recent proposition, introduced in 1905 by English biologist William Bateson (1861–1926), who rediscovered and spread in the scientific community Mendel’s principles of inheritance. Since the dawn of human civilization, the influence of heredity has been recognized, especially in agricultural crops and animal breeding [3].

The word *genetic* comes from the French *génétique*, formed from the Greek *genetikós*, “which refers to gender”, and that is the genitive case, transferred from the grammatical field to the biological one. *Gene*, from the German *Gen*, taken from the root of the Greek ‘génesis’ (generation). *Gènesis*, from the Latin *genĕsis*, is derived from the Greek génesis, an action noun of the verb *gígnomai,* “I generate”, meaning “origin” [4,5]. *Gen* was coined in 1905 by Wilhelm Ludvig Johannsen (1857–1927), who also coined the term genotype and phenotype [6,7]; Darwin used the term *pangenesis* in 1868 [8,9], later revived by Hugo De Vries (1848–1935) as Intracellular *Pangenesis* [10,11].

## 2. Hybridists and Hereditarists

Numerous considerations on anomalies passed along generations have been made since ancient times. In the modern era, among others, Ulisse Aldrovandi (1522–1605) and Fortunio Liceti (1577–1657) stood out.

The interest in inheritance found spur at the end of the 16th century with the observations of Prosper Alpinus (or Prospero Alpini, 1553–1616, physician and botanist, prefect of the medical garden, Padova University), who observed a sexual difference in date palm plants. This observation became relevant in the foundation of the Linnaean taxonomy system. Carolus Linnaeus himself (or Carl von Linné, 1707–1778) started in the eighteenth century the theory of plant sexuality. From these studies and through the impulse given by the Enlightenment to agricultural sciences, the current of thought of the *Vegetable Hybridists* developed. The studies on hybridization were the starting point of Mendel’s research.

Another group of scientists (*Hereditarists)* followed the *Preformationism* theory, which was very popular in the first half of the eighteenth century. The fundamental principle was already proposed by Anaxagoras (496–428 b.C.) and Democritus (460–370 b.C.) and then accepted in the *Corpus Hippocraticum*. According to the manuscript, the seed derived from all the districts of the adult body and is made up of small particles, which were capable of reproducing the whole body though the formation of the embryo [12].

Table 1 shows the scholars who have most contributed to studies on heredity before Gregor Mendel (Hynčice 1822–Brno 1884).

## 3. Inheritance and Genetics in Humans

**Figure 1:**
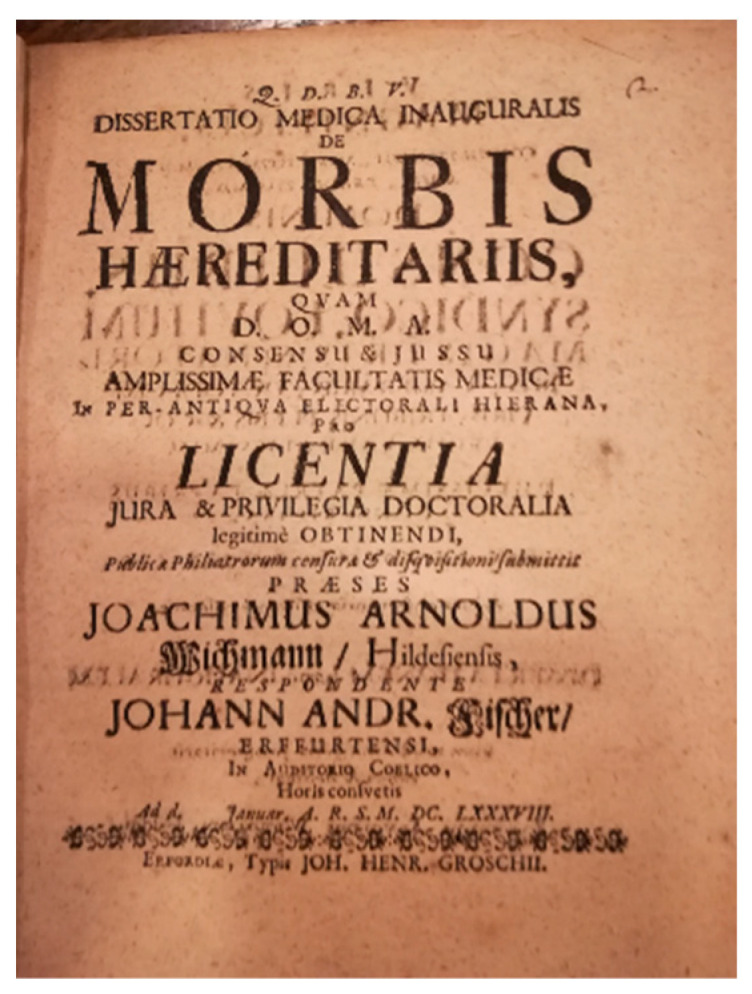
Frontispiece of Johann Andreas Fischer’s *Dissertatio Medica Inauguralis de Morbis Haereditariis*, 1688 (personal collection).

While most of the research was in the botanic field, among the *Hereditarists* there was also attention paid to human pathologies. In 1751, Pierre-Louis Moreau de Maupertuis (1698–1759) used the statistical analysis of the recurring polydactyly in the Ruhe family to demonstrate that (a) the phenomenon was not random but referred to a biological mechanism of character transmission that was still unknown; (b) the malformation was transmitted both maternally and paternally. The *Preformationism* idea was therefore amiss, in the *ovistic* and *animalculistic* versions. De Maupertuis suggested that the inheritance of characteristics was bound to “particles” in both germ cells. These particles would be responsible for the formation of different organs and structures in the baby [12]. Only in the second half of the 17th century hereditary diseases became the focus of some attention (see the *Dissertatio Medica Inauguralis* ‘*Morbis Haereditariis*’ by Johann Andreas Fischer at the Medical Faculty of Erfurt, January 1688. Frontispiece in Figure 1).

## 4. The Inheritance of Hearing Loss and Deafness

Apart from some sporadic descriptions, perhaps the first finding of a fairly large series of familial deafness was that of the island of Martha’s Vineyard [13]. For almost three centuries, a high rate of hereditary deafness appeared in the population of Martha’s Vineyard, an island in Dukes Country, southern Massachusetts, separated from Cape Cod by Vineyard Sound. In 1642, a group of migrants arrived from the county of Kent, in the south of England. The ancestor of most of the deaf was Jonathan Lambert, who moved there with his wife—who was not deaf—in 1694. When the migration virtually ceased, the endogamous community that was created contained a high incidence of hereditary deafness that persisted for over 200 years. Due to the isolation, a village sign language was developed, and it was once widely used in the island from the early 18th century to 1952. Martha’s Vineyard Sign Language was used by both deaf and hearing people in the community.

The GJB2 gene was causing deafness in Martha’s Vineyard. Today, we know hundreds of hereditary types of hearing loss (see *Hereditary Hearing Loss Homepage*) [14].

## 5. The Birth of Otology

«*Until the beginning of the 19th century, there were almost no measuring tools to determine and quantify the degree of hearing disability*» [15]. In the first part of the 19th century, new examination techniques became available, such as the otoscope with magnification and overall, functional hearing tests became possible. In 1802, Christian Heinrich Wolke (1741–1825) created the first ‘Akuometer’, and the main concepts of audiometry evolved. These innovations, together with the development of more adequate pathological knowledge, and therefore the possibility of a better diagnostic and therapeutic framework, and the improvement of surgical techniques allowed for the birth of new specialists dedicated to the treatment of otological pathologies, with the establishment of specialized hospitals (usually together with eye diseases).

The most significant contributions are those of the German, French, English, and Italian schools, as is reported in Table 2.

## 6. Deaf-Mutism

During the 19th century, there was a great debate on congenital deafness and consequent mutism. The prevalence was around 0.05–0.10% in Europe and a little less in the United States of America [16]. The existence of risk factors that influenced the frequency of deaf-mutism was already a topic of debate at that time [17]. However, Albert Eulenburg (1840–1917) notably addressed the issue of consanguinity while studying populations residing in mountainous regions, emphasizing the problem of *«close proximity of life and due to the often-deficient exchange with the outside world».* The inquiry considered consanguinity as a potential risk factor, exploring the idea that *«among those who enter into marriage, no other individual dispositions yet exist; inheritance, etc., for the procreation of deaf and dumb children»* [16].

By the end of the century, there was already a debated awareness of the existence of risk factors influencing the prevalence of deaf-mutism. However, clear causes of congenital deaf-mutism remained elusive.

## 7. Consanguinity

Various authors examined the incidence of deaf-mutes in consanguineous marriages [18,19,20,21,22,23].

In Germany, Richard Liebreich and Georg von Mayr reported a higher incidence of deaf-mutism among Jews compared to Christians (with a ratio ranging from 1:2 to 1:4). Additionally, albeit with a smaller difference, they noted a higher incidence among Protestants compared to Catholics [21,24]. These variations were linked to the prevalence of consanguineous marriages among Jews and the allowance of such marriages among Protestants, in contrast to the prohibition among Catholics. Schmaltz (1884) observed an increase in the frequency of deaf-mutism corresponding to the number of consanguineous spouses.

Eulenberg, recognizing the complexities involved, asserted that «*the question of the inheritance of congenital deaf-mutism is not truly resolved with all certainty through statistics*». He considered possibilities such as direct and indirect hereditary transmission: «*much more frequently, an indirect hereditary transmission of deaf-mutism can be demonstrated, either in the sense that the infirmity appears in ancestors and descendants, or monsters in the various lateral lines*»; further on he writes «*in addition to the above-mentioned more or less probable causes of congenital deaf-mutism, various authors also adduce a whole series of factors, especially important ones, such as the drunkenness of the parents, their mental illnesses, the large age difference, violent emotional excitations during pregnancy, etc., without it having until now been possible with statistics or accurate direct observations to even demonstrate the probability of an etiological link between these conditions and congenital deaf-mutism*» [16]. The question raised is whether consanguinity is the risk factor or the fact that «*among those who enter into marriage no other individual dispositions yet exist; inheritance*, etc. *for the procreation of deaf and dumb children*» [16].

An important indicator sufficient to raise suspicion of a congenital form of deaf-mutism was the presence of other conditions in relatives, such as additional cases of deaf-mutism or specific ocular diseases such as retinitis pigmentosasyndactyly, epilepsy, chorea, and cleft lip [21].

## 8. Heredity of Deafness

Wilde (1853) [25], Liebreich (1861) [19], and Uchermann (1869) [23] had found evidence that hearing impairment and deafness could be hereditary. Wilde wrote in 1853: «*That diseases of the ear are hereditary there is little doubt; and next to the congenitally deaf and dumb, I believe that nervous deafness is the most frequent form in which the disease is transmitted; but whether arising from some congenital peculiarity of the auditory nerve, which only becomes developed in after life, I cannot say. I know many cases in which mothers and daughters are deaf. I have also known several members of the same family and its collateral branches deaf of one ear. In the upper ranks of society, the disease is much more frequent in females than males*» [25].

An indirect familiar connection was described by different authors, with no clear direct inheritance [24,26]. A more frequent familiarity in the collateral line rather than the vertical line was described, besides that of a hypothetic recessive transmission.

Politzer wrote in 1882 that «*the most frequent causes of congenital deafness are: hereditary, including direct transmission from the parents as well as indirect transmission from forefathers and marriage between blood relatives*» [27]. This assertion stemmed from the research of Arthur Hartmann, who conducted studies in Berlin’s schools for deaf children [28]. Hartmann differentiated between the direct transmission of deafness from parent to child and indirect transmission, in which he observed a high incidence of consanguinity [23].

Giuseppe Gradenigo reported in 1887 not only the difference between “chronic catarrhal otitis media” and “otosclerosis”, but probably was the first to report on possible hereditary origin «*I must claim priority, for having, as early as 1887, asserted the very clear distinction between the two affections, and highlighted the degenerative hereditary nature of otosclerosis in contrast to the exogenous, inflammatory nature of chronic catarrhal otitis*» [29].

In the second edition of his *Lehrbuch der Ohrhenheilkunde*, published in 1887, Politzer clearly stated that deafness could be attributed to inheritance. He distinguished between direct or dominant inheritance and indirect or recessive inheritance, drawing on the research of another German author, Arthur Hartmann.

Holger Mygind [30], Uchermann, and Hartmann were among the early advocates for considering heredity as a significant factor. According to these authors, the consanguinity of parents only reinforces inheritance when it is present. Gradenigo (1903) asserted that «*heredity has a great influence in the production of congenital deaf-mutism*» and the presence of other cases of deaf-mutism in the family or retinitis pigmentosa serves as criteria supporting the congenital form [17]. Gradenigo also emphasized that «*in the case of ear diseases in general, the existence of other cases of deafness in the family is a valuable factor in differential diagnosis*» [31].

The first descriptions of syndromes involving hearing impairment or deafness emerged in the 19th century. Examples include Usher syndrome [21,32,33], branchio-oto-renal syndrome [34], Pendred syndrome [35], Treacher Collins syndrome [36,37], and osteogenesis imperfecta tarda [38]. While most hereditary hearing impairments are non-syndromic, more attention has been given to syndromic hearing disorders due to their distinguishable associated characteristics. Similarly, congenital forms of deafness have historically received more focus than late-onset hearing impairments.

## 9. The Audiometer and the Audiogram Phenotype

With the introduction of the audiometer in the late 1930s, describing and distinguishing non-syndromic forms of hearing impairment became more accessible. In 1955, Fisch described a relation between audiometric patterns and the etiology of perceptive deafness. He noted that «*a flat audiogram suggests rubella, a saucer-shaped audiogram kernicterus, a gently sloping audiogram with the high tones affected more than the low is often seen in dominant deafness, and a sharply sloping audiogram with a residual island of hearing in the low tones suggests autosomal recessive deafness*» [39].

Fraser (1970) reported on a substantial sample of 3534 individuals who had been “profoundly deaf from childhood,” describing numerous syndromic cases and focusing on causes based on “family history” [40]. He discussed the limited impact of discouraging deaf individuals from marrying each other in reducing the prevalence of genetically determined and recessive deafness. Fraser highlighted that such deafness is often not inherited from one generation to the next due to its association with numerous different genes. More frequently, the transmission of deafness from parent to child occurs when one of the parents has dominant deafness, regardless of whether the marriage partner is hearing or deaf [40].

Konigsmark (1971) emphasized the aid of five characteristics in the differential diagnosis of about 70 types of hereditary deafness in humans («*(1) The mode of genetic transmission, (2) the characteristics of the deafness, (3) the age of onset, (4) the sonic frequencies involved and (5) the associated abnormalities*»). These characteristics include the mode of genetic transmission, the characteristics of deafness, the age of onset, the sonic frequencies involved, and associated abnormalities [41]. The book “*Genetic and Metabolic Deafness*” by Bruce W. Konigsmark and Robert J. Gorlin, published in 1976 [42], along with subsequent editions, as well as McKusick’s “*Mendelian Inheritance in Man*” [43,44] and its online version [45], have been crucial reference sources for clinicians for decades.

## 10. Molecular Genetics and Epigenetics

The development of genetics, briefly reported in Table 3, was fundamental to identify new causes of hearing loss.

Since the mid-1900s, extensive efforts have been dedicated to comprehending the origins of both syndromic and non-syndromic hearing loss and identifying connections with specific audiological traits. In the last three decades, remarkable advances in molecular genetics have significantly contributed to our understanding of the causes and pathology of hearing impairment.

Linkage analyses in affected families have allowed researchers to successfully map the loci associated with hearing loss. Investigations of homozygosity have made it possible to identify autosomal recessive genes in consanguineous families. The first non-syndromic hearing loss locus was mapped in 1988 through the study of families with X-linked inheritance (DFNX2) [46,47]. It led to the identification of the POU3F4 gene in 1995 [48]. The first autosomal dominant locus (DFNA1) was linked to chromosome 5q31 in 1992 [49], and, a few years later, the DIAPH1 gene was identified [50]. The first autosomal recessive locus (DFNB1) was mapped in 1994 [51].

The first pathogenic variants of the GJB2 gene, encoding the protein connexin 26, the mutation of which are the cause of most forms of non-syndromic congenital deafness, was discovered in 1997 [52]. The identification of the GJB2 gene revolutionized the clinical approach to hearing loss, particularly for “isolated” deafness, which constituted the majority of cases labeled as “deafness of unknown cause.” Next-generation sequencing technologies have significantly improved the diagnostic rate for genetic hearing loss, allowing the detection of novel variants in known deafness-related genes and the identification of new genes associated with hearing disease.

Among the factors that cause syndromic and non-syndromic deafness, several mutations, which are in hundreds of genes, have been identified. Nowadays, approximately 206 non-syndromic hearing loss loci are known (78 autosomal dominant loci, 121 autosomal recessive loci, 7 X-linked loci, and one Y-linked locus). A total of 125 non-syndromic hearing loss-associated genes have been reported [14]. Finally, more than 400 syndromes that have hearing loss in their phenotypic presentation have been identified and described [53].

Scientists subsequently have turned their attention to epigenetics, a term coined by Conrad Waddington in 1942. Epigenetics is a branch of biology which studies heritable changes in gene expression that occur without alterations of the DNA sequence [54,55], regulating gene function through DNA and histone modifications, non-coding RNA expression, and chromatin remodeling. Five years after James Watson and Francis Crick first published the 3D structure of the DNA double helix [56]. In 1958, David Ledbetter Nanney [57] published a paper in which he used the term epigenetics to distinguish between different types of cellular control systems, trying to explain the relationships between genotype and phenotype [58,59]. Epigenetic regulation plays a vital role in maintaining genome stability, normal embryo development, and cell differentiation, and abnormalities can lead to various diseases, such as cancer [60] or type 2 diabetes.

Researchers in epigenetics and genetics collaborate closely to uncover the fundamental mechanisms of complex diseases, identifying novel therapeutic targets and prognostic indicators [61,62,63,64,65].

The significance of epigenetics in ear development is evident in human genetic disorders caused by mutations in genes involved in chromatin remodeling, DNA methylation, and histone modification. It is able to impact processes such as neurodevelopment [66], the development of external ear defects, and hearing loss. Vincent Michael Riccardi was probably the first scientist to consider the role of epigenetics in the genetic expression of genetic disorders in cranio-facial malformation hearing loss [67].

Epigenetic regulation influences various stages of cochlear hair cell (HC) life, such as differentiation, proliferation, and survival [68]. It contributes to HC regeneration, playing a critical role in ototoxic drug-induced hearing loss, noise-induced hearing loss, and D-gal-induced mimicking of presbycusis. Researchers are exploring compounds to regulate epigenetic modification as a potential avenue for alleviating acquired hearing loss. Epigenetic regulation is also crucial for inner ear HC regeneration, which offers an opportunity for hearing loss therapy [69,70].

Notably, environmental conditions, such as aging, lifestyle, infections, toxic exposure, and concurrent pathologies, can alter the epigenetic status, as reported by Gemmati and Collaborators [71]. Epigenetic markers may serve as indicators of disease, or contribute to the development of pathological conditions [72,73]. Inherited predispositions or gene mutations can shape an individual’s epigenetic landscape, potentially leading to various pathologies, including cancer, neurological diseases, pregnancy loss, and delayed wound healing [74,75,76]. Metal overload and epigenetic changes may also affect the cochlea or the sensorial epithelium, playing a role in various forms of sensorineural hearing loss. [77,78]. These facts were observed by the same group, describing how iron homeostasis genes predispose to one of the most difficult and complex forms of hearing loss, i.e., idiopathic sudden sensorineural hearing loss [79].

## 11. A Look at the Future: The “Omics Sciences” and Artificial Intelligence

Recently, there has been a lot of discussion about “Omics Sciences”, such as genomics, transcriptomics, proteomics, or metabolomics, and on the possible advantage of artificial intelligence (AI) for a better understanding of the causes and treatment of hearing loss [80]. The suffix “*-omics*” is used to address the objects of study in fields, such as the genome, proteome, transcriptome, or metabolome, respectively.

We will try to briefly summarize the state of the art of “Omics Sciences”.

### 11.1. Genomics

Genomics is the discipline that studies the genome of living beings. It deals with the structure, content, function, and evolution of the genome, utilizing bioinformatics to process and visualize the enormous amount of data that is produced [81].

Genomic technologies have transformed medicine in the post-genome era, promising personalized and precise healthcare based on DNA sequencing. The existing literature on this subject is extensive, with numerous reports continuously emerging, presenting new discoveries for a better understanding of various alterations [82,83] and potential gene therapies [84].

Before and immediately after the completion of the Human Genome Project, the primary obstacle to advancing precision medicine was the generation of DNA sequencing and genetic variant data. This challenge was particularly significant in the study of genetic hearing loss. The classification of genetic variants posed a major challenge in the post-genome era.

Richard Smith’s group evaluated and classified 876,139 hearing loss genetic variants; around 8100 variants were found to be pathogenic or likely pathogenic, 172,000 variants benign or likely benign, and more than 695,000 variants of uncertain significance [85]. The authors observed that over 96% of the coding variants were both rare and novel. They noted that the pathogenicity was influenced by factors such as minor allele frequency thresholds, variant effects, and protein domain considerations. The mutational landscape revealed intricate gene-specific variability, underscoring the importance of comprehending these nuances for enhanced accuracy in variant interpretation. This understanding is foundational for improving clinical decision-making and advancing our knowledge of deafness biology and treatment [86].

### 11.2. Proteomics

Proteomics is defined as the science that explores the interactions, function, composition, and structures of proteins and their cellular activities [87]. A proteome is a set of proteins produced in an organism, system, or biological context. Proteomics has proven effective in studying the inner ear. This approach aids in elucidating basic and pathologic mechanisms, diagnosing diseases, and treating hearing disorders [88,89,90,91,92,93]. Notably, recent research has highlighted the role of sophisticated glutamatergic ribbon synapses with afferent neurons to transmit auditory information to the brain. Furthermore, the multi-C2 domain protein otoferlin has been analyzed, linking defects in the coding Otof gene to auditory synaptopathy [94].

### 11.3. Transcriptomics

Transcriptomics, utilizing technologies such as bulk microarrays and RNA sequencing (RNA-Seq), provides a comprehensive view of the transcriptional landscape of auditory research [95,96]. Single-cell RNA-seq (scRNA-seq) technology has significantly contributed to the study of inner ear hearing, offering accurate transcriptome information for hair cell regeneration and hearing recovery. It also provides dimensional insights into the auditory organ, complementing other technologies mentioned above and holding potential for future clinical applications.

### 11.4. Metabolomics

Metabolomics, reflecting both genetic traits and environmental influences, plays a crucial role in understanding various forms of hearing impairment [97]. Recent studies have reported consistent data on the predictive role of serum metabolic profiles in sudden hearing loss [98], noise-induced hearing loss [99], and the identification of markers for ototoxicity [97,100]. Metabolomic investigations may pave the way for improved diagnostic strategies, personalized interventions, and targeted treatments, ultimately enhancing the clinical management of individuals affected by different causes of hearing loss [101,102].

### 11.5. Artificial Intelligence

Artificial Intelligence (AI) is not merely a topic of discussion, as the development of this technology is progressively expanding its possible applications [80]. AI is actively reshaping healthcare delivery. Specifically, deep learning (DL) and reinforcement learning (RL) methods are becoming indispensable factors in otolaryngology and communication sciences. While AI systems excel in specific areas like image and speech recognition, there is acknowledgment that the human brain still outperforms AI in many daily tasks [80]. The potential of AI to revolutionize healthcare, particularly in the field of otolaryngology, is increasingly recognized, with DL and RL methods playing crucial roles in achieving human-level or super-human AI systems [103]. To date, AI seems to be able to play an important role not only in the diagnosis of some complex forms disease [104,105,106] but also in sign language recognition and translation [107,108].

The enormous amount of data that can be obtained from “Omics Sciences” can be integrated and analyzed by artificial intelligence and the resulting digital transformation of systems science. But this is a story still largely to be written [109].

## Figures and Tables

**Table 1:** The major exponents of the two main currents of thought on heredity before Mendel’s laws.

Vegetable Hybridists	Hereditarists
Prospero Alpini (also known as Prosperus Alpinus, Prosper Alpinus, Prospero Alpinio) (Marostica 1553–Padua 1616) Adam Zaluziansky (Mnichov Hradiště 1558–Prague 1613) Nehemiah Grew (Atherstone 1641–London 1712) Rudolf Jakob Camerer (Tübingen 1665–Tübingen 1721) Carl von Linné (Råshult 1707–Uppsala 1778) Gottlieb Joseph Kölreuter (Sulz am Neckar 1733–Karlsruhe 1806) Thomas Andrew Knight (Wormsley 1759–London 1838) Augustin Sageret (Paris 1763–Paris 1851) Carl Friedrich von Gärtner (Göppingen 1772–Calw 1850) Henri Lecoq (Avesnes-sur-Helpe 1802–Clermont Ferrand 1871) Dominique Alexandre Godron (Hayange 1807–Nancy 1880) Charles-Victor Naudin (Autun 1815–Antibes 1899) Max Ernt Wichura (Neisse 1817–Berlin 1866)	William Wollaston (Coton–Clanford 1660–London 1724) Pierre-Louis Moreau de Maupertuis (Saint-Malo 1698–Basilea 1759) Georges-Louis Leclerc de Buffon (Montbard 1707–Paris1788) Charles Darwin (Shrewsbury 1809–Downe 1882) Karl Wilhelm von Nägeli (Kilchberg 1817–Munich 1891) Herbert Spencer (Derby 1820–Brighton 1903) Francis Galton (Sparkbrook 1822–Haslemere 1911) Thomas Henry Huxley (Ealing 1825–Eastbourn 1895)

**Table 2:** The major exponents of the birth and development of otology in the nineteenth century.

French School	German School
Antoine Saissy (1756–1822) Jean Marc Gaspard Itard (1775–1838) Gilbert Breschet (1784–1845) Pierre-Marie Flourens (1794–1867) Nicholas Deleau the Younger (1797–1862) Prosper Menière (1798–1862) Marcellin Emile Hubert-Valleroux (1812–1884) Alexandre Louis Paul Blanchet (1819–1867)	Ernst Heinrich Weber (1795–1878) Emil Huschke (1797–1858) Wilhelm Kramer (1801–1875) Eduard Schmalz (1801–1871) Carl Gustav Lincke (1804–1849) Gabriel Gustav Valentin (1810–1883) Hermann von Helmholtz (1821–1894) Ernst Reissner (1824–1878) Anton Friedrich von Tröltsch (1828–1867) Otto Friedrich Karl Deiters (1834–1863) Ádám Politzer (1835–1920) Hermann Schwartze (1837–1910)
**English school**	**Italian school**
William Robert Wilde (1773–1860) John Cunningham Saunders (1773–1810) William Wright (1773–1860) Samuel Akery (1785–1845) Henry Jones Shrapnell (1792–1834) James Yearsley (1805–1869) Joseph Toynbee (1815–1866)	Carlo Giovanni Brugnone (1741–1816) Alfonso Corti (1822–1876) Demetrio Bargellini (1823–1899) Giuseppe Gradenigo (1859–1926) Vittorio Grazzi (1849–1929) Vincenzo Cozzolino (1853–1911)

**Table 3:** The main stages of research that led to the discovery of the DNA double helix (1953).

Period	Discoveries
1866	Ernst Heinrich Haeckel (Potsdam 1834–Jena 1919), in 1866, proposed that the nucleus contained the factors responsible for the transmission of hereditary traits.
1869	Johann Friedrich Miescher (Basel 1844–Davos 1895) identified in 1869, inside the nuclei of human white blood cells, ‘nuclein’, the molecule now known as DNA
1870s	Walther Flemming (Schwerin 1843–Kiel 1905) in the 1870s described the morphology of a fibrous structure within the nucleus of cells, named ‘chromatin’, now known as chromosomes. By observing this chromatin, Flemming correctly worked out how chromosomes separate during cell division, also known as mitosis.
since 1879	Ludwig Karl Martin Leonhard Albrecht Kossel (Rostock 1853–Heidelberg 1927) made great progress in understanding the basic building blocks of nuclein. Kossel isolated the five nucleotide bases that are the building blocks of DNA and RNA: adenine, cytosine, guanine, thymine, and uracil. In 1881, Kossel identified nuclein as a nucleic acid and provided its present chemical name, deoxyribonucleic acid (DNA).
since the 1880s	Theodor Boveri (Bamberg 1862–Würzburg 1915) first presented the idea that the genetic material passed down from parent to child is within the chromosomes.
since 1905	Walter Stanborough Sutton (Utica 1877–Kansas City 1916) expanded on Theodor’s observation. He found it was possible to distinguish individual chromosomes undergoing meiosis in the testes of the grasshopper and identified the sex chromosome.
since 1905	Phoebus Aaron Theodor Levene (Sagor 1869–New York 1940), and his student J. A. Mandel, described a linear complex with a phosphoric acid and a base forming a subunit they called a mononucleotide, with two or more mononucleotides bound together to form what they called a polyphosphoric acid, or polynucleotide.
since 1910	Thomas H. Morgan (Lexington 1866–Pasadena 1945) and his students (Alfred Sturtevant, Calvin Bridges, Hermann Muller, and others), provided the proof for the chromosomal theory of heredity, genetic linkage, and chromosomal crossing over and non-disjunction.
1928	Frederick Griffith (Prescott 1879–London 1941) performed important studies on Bacterial transformation.
1944	Oswald Avery (Halifax 1877–Nashville1955). In 1944, he and his colleagues Maclyn McCarty and Colin MacLeod reported that the transforming substance, the genetic material of the cell, was DNA.
1950	Erwin Chargaff (Černivci 1905–New York 2002) found that in DNA, the ratios of adenine (A) to thymine (T) and guanine (G) to cytosine (C) are equal.
1951–1952	Maurice Hugh Frederick Wilkins (Pongaroa 1916–London 2004) and Rosalind Franklin (Kensington 1920–Chelsea 1958), at King’s College London, used X-ray diffraction to study the structure of DNA in solution. They found that DNA could take two forms: crystalline or A form, and paracrystalline or B form, as «big helix with several chains, phosphates on outside, phosphate–phosphate interhelical bonds, disrupted by water».
1953	J. D. Watson, F. H. C. Crick. Molecular structure of nucleic acids. A Structure for Deoxyribose Nucleic Acid. Nature 4356 April 25: 737-38, 1953 M. H. F. Wilkins, A. R. Stokes, H. R. Wilson. Molecular Structure of Deoxypentose Nucleic Acids. Nature 4356 April 25: 738-40, 1953

## Data Availability

Data sharing not applicable.
